# Cognitive Adaptation Training Provided to Chronically Hospitalized Patients with Schizophrenia in The Netherlands: Two Case Reports

**DOI:** 10.1155/2012/596162

**Published:** 2012-10-02

**Authors:** Piotr J. Quee, Harald Schneider, Saskia van Slogteren, Durk Wiersma, Richard Bruggeman, Dawn I. Velligan

**Affiliations:** ^1^Department of Psychiatry and Rob Giel Research Center, University Medical Center Groningen, University of Groningen, P.O. Box 30.001, 9700 RB Groningen, The Netherlands; ^2^Department of Rehabilitation, Lentis Center for Mental Health, P.O. Box 128, 9470 AC Zuidlaren, The Netherlands; ^3^Division of Schizophrenia and Related Disorders, Department of Psychiatry, University of Texas Health Science Center, Mail Stop 7797, San Antonio, TX 78229-3900, USA

## Abstract

Cognitive adaptation training (CAT) improves functional outcome in outpatients with schizophrenia living in the United States of America. The efficacy of CAT has never been demonstrated for patients living in a residential facility. We describe how CAT was delivered to two chronically hospitalized patients with schizophrenia living in The Netherlands. CAT was delivered for 8 months, and consisted of weekly home visits by a psychiatric nurse. Both patients improved on measures of functional outcome used in the US studies. These results indicate that CAT may improve outcomes, even in patients that have been hospitalized for several years.

## 1. Introduction

Cognitive impairments in schizophrenia substantially contribute to long-term functional disability [1]. Cognitive adaptation training (CAT) is a home-delivered psychosocial intervention, that aims to improve functional outcomes by reducing the effect of cognitive impairment on the patient's life [2]. CAT treatment plans are based on a neuropsychological evaluation and an evaluation of the patients' functioning and living environment. For the former, the Modified Card Sorting Task (MCST) and Controlled Oral Word Association Task (COWAT) are used to determine whether the patients' level of cognitive/executive function is fair or poor, compared to other patients with schizophrenia [3, 4]. Additional cognitive tasks can be administered to make the evaluation more comprehensive. The Frontal Systems Behavior Scale (FrSBe) is administered to assess whether overt behaviour is characterized by apathy (poverty in speech, initiation problems), disinhibition (disorganization, distractibility), or a combination of these [5]. Finally, the Environmental and Functional Assessment (EFA) is used to evaluate the patients' independent living skills in their living environment, presence of safety hazards (e.g., frayed electrical cords), and availability of needed supplies (e.g., detergent, toothpaste) [6].

We then share the results of the assessments with the patient. Specific problem areas the patient wants to work on are prioritized. CAT has proven to successfully improve functional outcomes for patients living in the United States of America [7–9]. A recent study carried out in Denmark did not find an additive effect of CAT when added to their standard treatment [10]. However, it can be suggested that their study was not carried out in line with the earlier CAT studies [11]. In the current paper, we describe how CAT was delivered by a psychiatric nurse to two patients from The Netherlands, living in a residential facility. CAT was provided for 8 months for 45 minutes weekly. Thereafter, the CAT interventions were transferred to the patients' case manager. The psychiatric nurse received weekly supervision from a psychologist (P.Q.). The patients described in this paper were participating in a CAT pilot study, which was approved by a local Medical Ethics Committee.

## 2. Case  1

Mr. C. is 54 years old and hospitalized for over 20 years, living in a group home with 6 other patients. Clinical symptoms included auditory hallucinations, social withdrawal, anxiety, and stereotypical behaviours. Mr. C.'s performance on neuropsychological testing indicated fair executive functioning. The FrSBe revealed behavioural apathy. Results of the assessments of executive function and clinical symptoms are given in Table 1. Mr. C. had ceased participating in work-related activities at the residential facility five years ago. Currently, he spent most of his time in his small room, which consisted of his bed, desk, sink, and clothing cabinet. Interests he enjoyed in the past but was not currently pursuing included reading comics and electronic music. The EFA revealed a very restricted eating behaviour. Mr. C. attributed this to his voices, causing him not to eat meals nor to brush teeth. He was unable to perform a variety of household chores, including doing laundry. Mr. C. had many clothes in his cabinet, but he preferred wearing two identical clothing sets. The staff visited him every day for his medication and meal delivery, and the patient experienced them as supportive. His case manager visited him once a week for 30 minutes. Mr. C. was prescribed antipsychotic drugs (Leponex) and anticholinergics (Akineton).

Personal hygiene was the first area that the patient agreed to work on. The CAT therapist gave him a comic on brushing teeth, to make the discussion of the activity easier. Mr. C. admitted the relevance of brushing teeth but was afraid to throw up from the taste of the toothpaste. A soft brush and an own desired toothpaste flavour (strawberry) were provided on trial. Mr. C. began to carry out the activity on a daily basis. A checklist containing brushing teeth every day was used to compensate for apathy. Later on, other hygiene activities were added to this list. Initially, we reinforced improvement in hygiene, by providing a new comic. Mr. C. did not have a number of household items (e.g., soap, deodorant), so the CAT therapist provided them. 

Starting from the second month of CAT, sessions also consisted of activities outside the home. A map of the village was provided to improve Mr. C's orientation to the neighbourhood. The CAT therapist gave him the task of finding a specific location (e.g., activity center) on the map, and then Mr. C. showed her the route. After visiting a number of activity centres, Mr. C. was more enthusiastic about these services and asked to participate in wood crafting 8 hours a week. One month later, Mr. C. asked to work another 4 hours per week at a framing center. The tasks at the latter location were more varied and cognitively demanding, and the new colleagues of Mr. C. were less impaired than he was. Therefore, the CAT therapist visited the workplace and worked with staff to adapt the working environment by giving Mr. C. only one type of task at a time. This enabled him to successfully complete assigned tasks. 

Every morning Mr. C. was scheduled for work; a voice cue from a personalized alarm prompted him to wake up. This alarm was also used to prompt him to take his medication, which was previously administered by staff. This made Mr. C. independent in taking his medication for the first time in 20 years. Due to the physical nature of his job he began to experience physical complaints. Therefore, during work, he was instructed by the staff when to take a break. After a few weeks, Mr. C. was able to initiate this himself. In addition, swimming was introduced as a new physical exercise with the goal to reduce muscle stiffness. He was reminded to engage in this activity by adding it to his calendar.

By the end of the treatment, Mr. C. had increased his participation in work-related activities from 0 days to 4 partial days per week. The staff no longer observed a body odor, and Mr. C. was more easygoing in personal contact. The interventions were explained and gradually transferred to his case manager after 6 months. After 8 months, the case manager visited Mr. C. to evaluate his checklist and to monitor his medication adherence and hygiene and participation in work-related activities all of which remained substantially improved from baseline.

## 3. Case  2

Mr. J. is a 24-year-old male with early-onset schizophrenia, hospitalized for 4 years. Current clinical symptoms included auditory hallucinations, disorganization, and blunted affect. Neuropsychological task performance revealed fair executive functioning. The FrSBe revealed behavioural disinhibition. Results of the assessments of executive function and clinical symptoms are given in Table 1. Work-related activities consisted of parttime sheltered work at the kitchen of the clinic. He appreciated social contact but did not have many friends, as many group members were older. The EFA revealed problems with demonstration of several functional tasks, including using kitchen utensils. He spent much of his time idle. Unless reminders were given by the staff, he would miss doctor appointments and fail to take care for his personal hygiene. His living area was cluttered. There were spills on the floor, and his belongings were disorganized. Mr. J. was also diagnosed with a severe (grade V) vesicoureteral reflux in both kidneys. As a result of this pathology, he needed to perform multiple self-catheterizations a day. At least once every other day, he forgot to do this. Mr. J. lived at a group home together with 26 other patients. Here he had his own apartment on the first floor, which included a bathroom and kitchen. A staff room was situated at the ground floor, where medication and meals were provided. Staff was present all day. His case manager visited him once a week for 30 minutes. Mr. J. was prescribed antipsychotic drugs (Leponex, Abilify), anticholinergics (Artane), selective beta blockers (Atenolol), antiepileptics (Depakine), and benzodiazepines (Oxazepam).

Interventions started with reorganizing belongings. Most items strewn around consisted of clothing or paperwork. Mr. J. was provided with a file folder for his paperwork. In his clothing cabinet, we attached labels to shelves for several clothing items (e.g., socks, t-shirts). In a similar way, we organized his DVDs, comics, and computer games. Next, he was provided with a weekly checklist, with activities that he wanted to carry out more frequently. The first items on this list contained catheterizing and brushing teeth. 

We attempted to improve social contact by introducing Mr. J. to other patients with similar interests. A young patient living at a neighbouring apartment reacted positively to this. This patient and Mr. J. agreed to meet on a weekly basis and cook a meal. The CAT therapist trained Mr. J. in meal preparation. In the first session, he was provided with a recipe, consisting of easy sentences for every subtask which Mr. J. followed. The therapist intervened only when necessary. Mr. J. needed help with cooking times and measuring quantities. Since Mr. J. did not have a timer and a measuring cup, these were provided. The next session was used to rehearse the same meal, using the supports provided. After three sessions, Mr. J. was able to prepare this meal using the instructions of the recipe and without interventions from the CAT therapist. In a similar way, the CAT therapist and Mr. J. worked on a second and third recipe. 

Mr. J. needed more support to carry out the activities on his checklist more regularly. A voice-cue alarm was put in his room to remind him of brushing his teeth at the end of the day. Gradually, the voice-cue alarm was used to prompt him carrying out a larger repertoire of activities (e.g., taking a shower, going to sleep). This intervention was explained to nursing staff members of his group, and they were asked to evaluate the items on his checklist at the end of the evening. Such evaluations started with “let's look at your checklist together.” For catheterization, an important event was appearing on his checklist that he had to carry out multiple times a day Mr. J. was also provided with an alarm-watch. With the help of the CAT therapist, Mr. J. wrote down tasks and appointments that occurred less often than daily on a calendar that he and the therapist placed on the bathroom door. These interventions increased the target behaviors.

After 6 months of treatment, Mr. J. visited his neighbour multiple times a week, he took a shower at least every other day, and he spent more hours on leisure activities. He was also able to increase his participation in work-related activity. His case manager became more closely involved in the process. She created brief weekly contact to discuss the interventions with him, and this made it possible to sustain Mr. J.'s improved functioning on a longer term.

## 4. Conclusions

**Figure 1:**
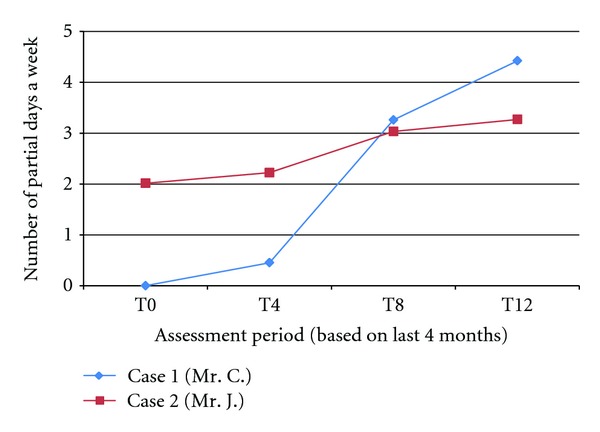
Participation in work-related activities for the two cases.

The current case reports describe how CAT was delivered to chronically hospitalized patients with schizophrenia in the Netherlands. This is a different population than treated with CAT in efficacy studies in the US and Denmark, where CAT participants lived in the community or in minimally supervised board and care homes [7–10]. Chronically institutionalized patients may be more treatment resistant. Moreover, these individuals may have the support of involved staff to prompt and cue activities. The extent to which CAT will be effective in this group of individuals is unclear. However, the case reports provide support for the efficacy of CAT in this group. We found improvements on the same measure of functional outcome as in the US studies (Table 2) and on work-related activities which were sustained over a longer time (Figure 1). CAT differs from other psychosocial interventions, in that it uses a comprehensive assessment to create supportive environments for the individual. The way in which these supports are established differs from individual to individual, dependent on behavioral style and level of cognitive/executive function. This customized intervention shows promise for improving functional outcome. Randomized, controlled studies in the future are needed to systematically test the efficacy of CAT in this chronically hospitalized population. It may be that CAT could be utilized to move individuals with schizophrenia to more independence in daily living.

## Figures and Tables

**Table 1:** Baseline characteristics for the two cases.

	Case 1 (Mr. C.)	Case 2 (Mr. J.)
Executive function,		
MCST^a^,		
Categories	1	4
Perseverations	18	2
COWAT^b^	26	8
FrSBe^c^,		
Apathy	48	32
Disinhibition	37	38

Clinical Symptoms (PANSS)^d^,		
Positive	14	15
Negative	29	28
General	41	33

^a^MCST: Modified Card Sorting Test [3]; ^b^COWAT: Controlled Oral Word Association Task—Dutch version [4]; ^c^FrSBe: Frontal Systems Behavior Scale—informant version [5]; ^d^PANSS: Positive and Negative Syndrome Scale [12], scaled score based on the last week. For the MCST perseverations, FrSBe, and PANSS, higher scores indicate poorer outcomes.

**Table 2:** Scores on the measures of functional outcome for the two cases.

	Case 1 (Mr. C.)	Case 2 (Mr. J.)
MCAS^a^		
T0	44	53
T4	48	54
T8	52	58

NSA^b^		
T0	23	18
T4	21	18
T8	20	15

^a^MCAS: Multnomah Community Ability Scale [13], total score based on last 3 months (higher scores indicate better outcomes); ^b^NSA: Negative Symptom Assessment [14]—motivation subscale based on last week (higher scores indicate poorer outcomes).
